# Cyclic AMP induces apoptosis in multiple myeloma cells and inhibits tumor development in a mouse myeloma model

**DOI:** 10.1186/1471-2407-11-301

**Published:** 2011-07-18

**Authors:** Virginie Follin-Arbelet, Peter O Hofgaard, Harald Hauglin, Soheil Naderi, Anders Sundan, Rune Blomhoff, Bjarne Bogen, Heidi K Blomhoff

**Affiliations:** 1 Department of Biochemistry, Institute of Basic Medical Sciences, University of Oslo, Oslo, Norway; 2 Centre for Immune Regulation, Institute of Immunology, University of Oslo and Oslo University Hospital, Rikshospitalet, Oslo, Norway; 3 Department of Nutrition, Institute of Basic Medical Sciences, University of Oslo, Oslo, Norway; 4 Department of Cancer Research and Molecular Medicine, NTNU, Trondheim, Norway

## Abstract

**Background:**

Multiple myeloma is an incurable disease requiring the development of effective therapies which can be used clinically. We have elucidated the potential for manipulating the cAMP signaling pathway as a target for inhibiting the growth of multiple myeloma cells.

**Methods:**

As a model system, we primarily used the murine multiple myeloma cell line MOPC315 which can be grown both *in vivo *and *in vitro*. Human multiple myeloma cell lines U266, INA-6 and the B-cell precursor acute lymphoblastic leukemia cell line Reh were used only for *in vitro *studies. Cell death was assessed by flow cytometry and western blot analysis after treatment with cAMP elevating agents (forskolin, prostaglandin E2 and rolipram) and cAMP analogs. We followed tumor growth *in vivo *after forskolin treatment by imaging DsRed-labelled MOPC315 cells transplanted subcutaneously in BALB/c nude mice.

**Results:**

In contrast to the effect on Reh cells, 50 μM forskolin more than tripled the death of MOPC315 cells after 24 h *in vitro*. Forskolin induced cell death to a similar extent in the human myeloma cell lines U266 and INA-6. cAMP-mediated cell death had all the typical hallmarks of apoptosis, including changes in the mitochondrial membrane potential and cleavage of caspase 3, caspase 9 and PARP. Forskolin also inhibited the growth of multiple myeloma cells in a mouse model *in vivo*.

**Conclusions:**

Elevation of intracellular levels of cAMP kills multiple myeloma cells *in vitro *and inhibits development of multiple myeloma *in vivo*. This strongly suggests that compounds activating the cAMP signaling pathway may be useful in the field of multiple myeloma.

## Background

Multiple myeloma (MM) is a B-cell malignancy characterized by accumulation of plasma cells in the bone marrow, osteolytic bone lesions, and immunodeficiency [1]. It accounts for ~10% of hematological malignancies [2] with a median survival of 4 years [3]. Despite the progress made the last decades in the development of new therapies, multiple myeloma remains an incurable disease for which a constant search for new treatment strategies must continue.

Cyclic adenosine monophosphate (cAMP) is an intracellular messenger formed in response to diverse extracellular stimuli including hormones or neurotransmitters. It is generated from ATP by adenylyl cyclases, and is degraded by phosphodiesterases (PDE) into adenosine-5'-monophosphate. The main targets of cAMP are protein kinase A (PKA) [4], cAMP-gated ion channels [5] and exchange proteins directly activated by cAMP (EPAC) [6]. cAMP affects numerous cellular processes, such as cell differentiation, cell cycle progression and apoptosis, both in a PKA-dependent and PKA-independent manner [7-9]. In many cancer tissues and cell lines, alterations in cAMP signaling pathway including changes in intracellular levels of cAMP [10,11] and PKA isoforms ratio switch [12-15], have been observed. Consequently, there is a growing interest in manipulating the cAMP signaling pathway as a strategy for the treatment of cancer, and in particular a renewed interest for the potential of combining PDE inhibitors and glucocorticoids for treatment of hematological malignancies [16].

We have previously shown that cAMP blocks the G1/S phase transition and DNA synthesis in lymphoid cells [17-19]. More recently, we demonstrated that elevation of intracellular cAMP alone has no effect on cell death in B-cell precursor acute lymphoblastic leukemia (BCP-ALL) cells, but that it prevents apoptosis and accumulation of p53 in the cells subjected to γ-irradiation (γ-IR) [20]. In the present paper, we have explored the role of cAMP in multiple myeloma by primarily using the multiple myeloma cell line MOPC315. This cell line was chosen as it is a suitable mouse model [21,22] for studying the effect of cAMP on development of multiple myeloma *in vivo*. Elevation of intracellular levels of cAMP in the multiple myeloma cells did not prevent γ-IR-mediated death of the cells *in vitro*, but interestingly, cAMP alone efficiently killed the myeloma cells. More importantly, we could demonstrate that cAMP prevents the growth of multiple myeloma cells *in vivo*.

## Methods

### Chemicals, Antibodies

Forskolin and rolipram (Sigma; Saint Louis, MO, USA) were diluted in dimethyl sulfoxide (DMSO), 8CPT-cAMP (Biolog, Bremen, Germany) was diluted in distilled water, whereas prostaglandin E_2 _(Cayman, Ann Arbor, MI, USA) was diluted in ethanol. Propidium iodide, DMSO, saponin, paraformaldehyde and bovine serum albumin (BSA) were purchased from Sigma. The cationic fluorescent carbocyanine dye 5,5',6,6'-Tetrachloro-1,1',3,3' -tetraethylbenzimidazolylcarbocyanine iodide (JC-1) was from Calbiochem (San Diego, CA, USA).

Antibodies against caspase 3 (8G10), caspase 9 (the mouse-specific 9504 and the human-specific 9502) and PARP were purchased from Cell Signaling Technologies (Danvers, MA, USA). P53 (fl393) antibody was purchased from Santa Cruz Biotechnology (Fremont, CA, USA). Antibody against GAPDH (Sigma) was used as a loading control. Anti-goat and anti-mouse HRP-conjugated secondary antibodies were purchased from Bio-Rad (Hercules, CA, USA).

### Irradiation of the cells

Cells were irradiated using a ^137^Cs source at 4.3 Gy/min.

### Cell lines and cell culture

The BCP-ALL cell line Reh [23] was cultured as previously described [19]. The transplantable BALB/c mineral oil-induced plasmacytoma cell line, MOPC315 [21], was used to generate a subline, MOPC315.4, that grew well in vitro and in vivo [24]. A subline of MOPC315.4, MOPC315.BM (Bogen et al., unpublished), was used for the present experiments. Some experiments employed MOPC315.BM labeled with the fluorescent protein DsRed. For simplicity, the MOPC315.BM subline will be referred to as MOPC315 throughout the paper. The cells were cultured in vitro in RPMI 1640 (Invitrogen, Paisley, UK) containing 2 mM L-glutamine, supplemented with MEM non essential amino acid (Sigma), 1 mM sodium pyruvate (Sigma), 50 μM monothioglycerol (Sigma), 12 μg/ml gentamycin (Sigma) and 10% heat-inactivated FBS (Lonza, Verviers, Belgium). The human multiple myeloma cell line, INA-6 cells, was a kind gift from Dr. M. Gramatzki (Erlangen, Germany) and were cultured in RPMI 1640 (Invitrogen) containing 2 mM L-glutamine (Invitrogen), supplemented with 1 ng/ml Il-6 (Invitrogen), 12 μg/ml gentamycin (Sigma) and 10% heat-inactivated FBS (Lonza). The U266 cell line was purchased from the American Type Culture Collection (ATCC, Manassas, VA, USA) and was cultured in RPMI 1640 (Invitrogen) containing 2 mM L-glutamine (Invitrogen), supplemented with 15% heat-inactivated FBS (Sigma), 100 U/ml penicillin (Invitrogen), and 100 μg/ml streptomycin (Invitrogen).

### Flow cytometry

Flow cytometry analysis was performed on a FACS Calibur (Becton-Dickinson). For determination of cell viability by exclusion of propidium iodide (PI), 500 μl of cell culture were incubated with 20 μg/ml PI for 10 min at room temperature prior to analysis. The cationic fluorescent carbocyanine dye, 5,5',6,6'-tetrachloro-1,1',3,3' -tetraethylbenzimidazolylcarbocyanine iodide (JC-1) was used to assess changes in the mitochondrial membrane potential (ΔΨm) observed in apoptotic cells. Cells were incubated for 15 min at 37°C with 15 μg/ml JC-1 before analysis. For determination of apoptotic cells, TUNEL assays were performed by using an In Situ Cell Death Detection Kit, Fluorescein from Roche (Mannheim, Germany). Briefly, cells were washed in ice cold PBS before being fixed with 4% paraformaldehyde and permeabilized with 0,1% saponin. Cells were washed in ice cold PBS before incubation in the TUNEL reaction mix for 1 h at 37°C. After washing the cells 3 times, the cells were analyzed by flow cytometry.

### Immunoblot analysis

Cells were lysed in RIPA buffer (50 mM Tris [pH7.5], 150 mM NaCl, 1% NP-40, 0.1% SDS, 0.5 mM EDTA, 50 mM NaF, 10 mM β-glycerophosphate, 1 mM Na3VO4, 0.2 mM phenylmethylsulfonyl fluoride [PMSF], 10 μg/ml leupeptin, and 0.5% aprotinin) and an equal amount of proteins (50 μg) was separated by SDS-PAGE (Bio-Rad) electrophoresis. After transfer to a nitrocellulose membrane (GE Healthcare) using a semidry transfer cell (Bio-Rad), proteins were detected by standard immunoblotting procedures. In brief, the nitrocellulose membranes were washed in Tris buffered saline and 0.1% Tween (TBST) and incubated in blocking solution (5% non-fat dry milk in TBST or 5% BSA in TBST) at room temperature. After washing, the membranes were incubated overnight at 4°C with primary antibodies diluted in blocking solution. After washing in TBST, the membranes were incubated for 1 h with HRP-conjugated secondary antibody diluted in blocking solution, followed by a final washing at room temperature. Immunoreactive proteins were visualized with the enhanced chemiluminescence detection system (ECL, Amersham Pharmacia Biotech, UK) or the SuperSignal^® ^west Dura Extended Duration substrate (Thermo Scientific, Rockford, IL, USA) according to the manufacturer's protocol.

### Mouse model for multiple myeloma

Adult BALB/c nude mice (purchased from Charles River, Germany) were injected subcutaneously in the interscapular region with 5 × 10^5 ^tumor MOPC315.DsRed cells suspended in 100 μL PBS. Two days after injection of the cells, 5 mice were injected intraperitoneally with 4-5 mg/kg forskolin diluted in a PBS/DMSO solution (15:0.1), and 5 mice were injected with the vehicle. In a separate experiment, forskolin (or vehicle) was injected 3 times on days 2, 4 and 6. Tumor growth was followed daily by palpation and imaging. Mice with tumor diameters of 15-20 mm were killed by cervical dislocation. The study was approved by the National Committee for Animal Experiments.

### In vivo imaging of mice

Mice were anaesthetized with 2.5% isoflurane (Baxter As, Norway). Immediately afterwards, they were placed in a light-sealed imaging chamber and kept anaesthetized throughout the imaging period.

Images were acquired using a combination of excitation (30 nm passband) and emission (20 nm passband) filters on an IVIS Spectrum Imaging System (Caliper Life Sciences). The following spectral channels were used (excitation:emission center wavelength in nm): 465:540, 465:580, 535:600 and 570:620. Spectral images were recorded in units of photons/second/cm^2^/sr and imported as 32 bit floating point TIFF files into Mathematica 5.2 (Wolfram Research) for further processing. Images were scaled with an excitation light correction factor [25] yielding normalized fluorescence efficiency (NFE) images for further processing. Background reference autofluorescence spectrum was recorded from the interscapular region on day 0 before MOPC315 injection. A reference MOPC315.DsRed spectrum was determined from a region containing a localized tumor (day 5) with the reference autofluorescence subtracted. MOPC315.DsRed specific signal was determined by linear (pseudo-inverse) unmixing [26], yielding DsRed fluorescence maps, which were thresholded, intensity color-coded and overlaid a white light illuminated image. Quantification of MOPC315.DsRed fluorescence was done by computing the total DsRed fluorescence for above-threshold pixels for each animal.

### Statistical analysis

The paired-samples t-test was applied to check the significance in cell line experiments, using the PASW Statistic 18 software for windows. In all the figures, histograms show mean values of the indicated number of experiments, with error bars corresponding to SEM values. For *in vivo *experiments, the Wilcoxon signed-rank test was used to determine significant differences between 2 groups of mice.

## Results

### Elevation of cAMP levels by forskolin induces death of multiple myeloma cells

**Figure 1:**
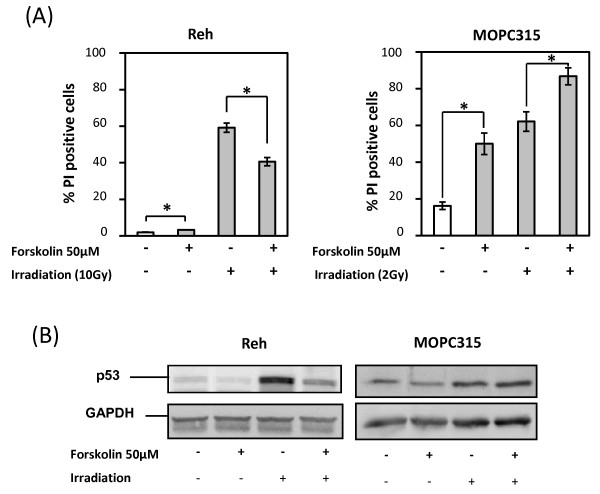
**Effect of forskolin on DNA-damaged induced cell death**. Vehicle or 50 μM forskolin was added to cell cultures 30 min prior to exposure of Reh cells and MOPC315 cells to 10Gy and 2Gy, respectively. (A) 24 h after irradiation, cell death was assessed by PI staining. The results represent the mean values +/- SEM of 3 (Reh cells) or 6 (MOPC315 cells) experiments respectively. *P < 0.05 relative to cells treated with vehicle. (B) 4 h after irradiation, cells were harvested and examined for p53 and GAPDH expression by Western blot analysis.

We have previously shown that in B-cell precursor acute lymphoblastic leukemia (BCP-ALL) cells, elevated intracellular levels of cAMP prevent apoptosis induced by a variety of DNA-damaging cytotoxic agents, including ionizing radiation (IR). We have also demonstrated that destabilization of p53 is a key feature in this process [20]. Now we have compared the effects of the adenylyl cyclase-activating diterpine forskolin [27] on IR-mediated cell death of the BCP-ALL cell line Reh and the plasmacytoma cell line MOPC315. Figure 1A (left panel) shows that forskolin (50 μM) alone had no effect on the viability of the Reh cells, but unsurprisingly prevented IR-induced apoptosis, as measured 24 h later. In sharp contrast, forskolin did not prevent IR-mediated death of the myeloma cells (Figure 1A, right panel), but rather potentiated the effect of irradiation. More intriguingly, after 24 hours of sole forskolin treatment, the percentage of dead MOPC315 cells increased from ~16% to ~50%.

To confirm the differential effect of forskolin on IR-treated Reh cells and MOPC315 cells, the effect of elevating cAMP on p53 expression was analyzed. In accordance with our previous result [20], forskolin prevented the IR-induced stabilization of p53 in Reh cells (Figure 1B). In contrast forskolin had no effect on p53 induced by IR in MOPC315 cells. Importantly, forskolin alone decreased the p53 levels in MOPC315 cells, indicating that induction of p53 is not involved in cAMP-mediated cell death of MOPC315 cells. Myeloma cells were notably more sensitive to irradiation than Reh cells; only 2 Gy was used to obtain similar death in MOPC315 cells compared to 10 Gy in Reh cells.

### Dose- and time-dependent effects of forskolin on death of MOPC315 cells are mediated via cAMP

**Figure 2:**
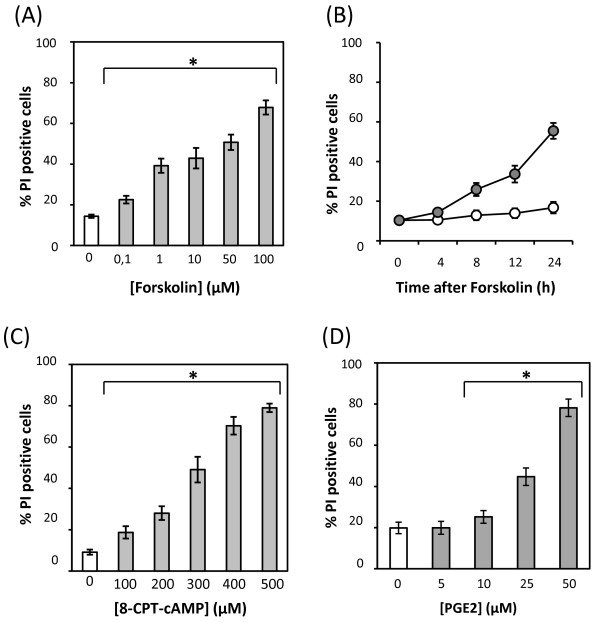
**cAMP induces cell death in MOPC315 cells**. MOPC315 cells were incubated for 24 h with the indicated concentrations of forskolin (A), with vehicle (open circles) or 50 μM forskolin (closed circles) for the indicated incubation times (B), or with the stated concentrations of 8-CPT-cAMP (C) or prostaglandin E2 (D) for 24 h. Cell death was measured by PI-staining, and the results are presented as percentage of PI-positive cells +/-SEM. A,C and D, n = 4; B, n = 3. *P < 0.05 relative to cells treated with vehicle.

MOPC315 cells were treated for 24 h with increasing doses of forskolin, or with 50 μM of forskolin, at various time points. Cell death was measured by incorporation of PI. Forskolin induced death of MOPC315 cells was both dose- and time-dependent (Figure 2A and 2B, respectively). Death occurred at doses as low as 0.1 μM forskolin, and statistically significant death could be detected already after 8 h with 50 μM forskolin.

In addition to the activation of adenylyl cyclase, forskolin has been reported to modulate other cellular processes, such as ion channels [28,29]. We tested the effect of other cAMP increasing agents to verify that forskolin-induced cell death was mediated by intracellular accumulation of cAMP. This included the cell membrane permeable cAMP analog 8-chlorophenylthio-cAMP (8CPT-cAMP) and prostaglandin E2 (PGE2), which increases intracellular levels of cAMP through the activation G-protein-coupled receptors [30,31]. MOPC315 cells were killed in a dose-dependent manner by 8CPT-cAMP or PGE2 (Figure 2C and 2D, respectively), supporting the notion that forskolin kills the MOPC315 cells via induction of cAMP.

### cAMP induces death of human multiple myeloma cells

**Figure 3:**
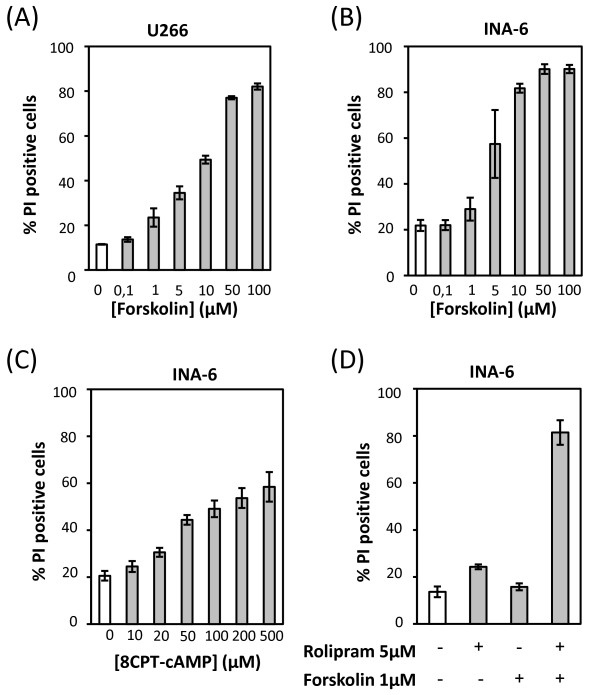
**cAMP induces cell death in human multiple myeloma cell line**. U266 cells and INA-6 cells were incubated for 24 h with the stated doses of forskolin (A and B) or 8-CPT-cAMP(C) or rolipram and forskolin(D). Cell death was assessed by PI staining. The results are presented as the mean values +/-SEM of 3 experiments.

To verify that the killing of murine multiple myeloma cells by cAMP was applicable to human myeloma cells, the human multiple myeloma cell line U266 and the IL-6-dependent human myeloma cell line INA-6 were included. The cells were treated for 24 h with increasing doses of forskolin or 8CPT-cAMP, and cell death was assessed by PI incorporation. Both human multiple myeloma cell lines were sensitive to intracellular elevation of cAMP (Figure 3A, B and 3C), indicating that elevation of intracellular levels of cAMP indeed also induces cell death in human multiple myeloma cells. By using a cAMP Biotrack enzymeimmunoassay (EIA), we verified that forskolin increased the intracellular cAMP concentrations in these two cell lines (data not shown). To further confirm the involvement of the cAMP signaling pathway in killing multiple myeloma cells, INA-6 cells were treated for 24 hours with a low dose of forskolin in combination with rolipram, an inhibitor of PDE4. Rolipram or a low dose of forskolin alone induced little or no cell death, whereas a combination of the two compounds markedly increased cell death (Figure 3D).

### cAMP induces apoptotic cell death in multiple myeloma cells

**Figure 4:**
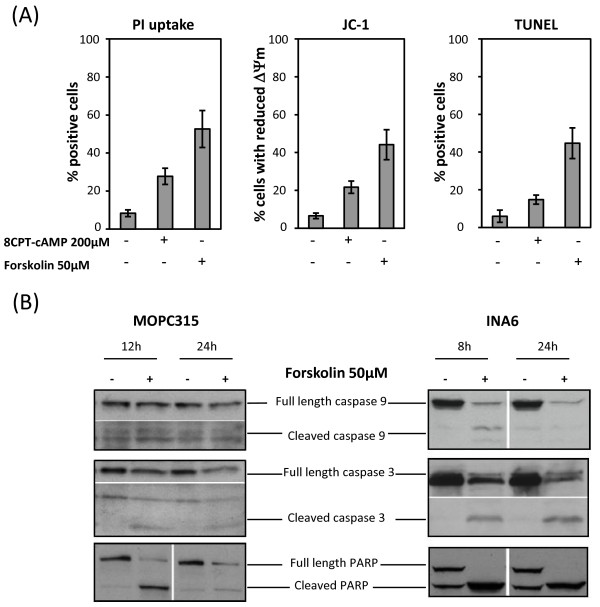
**cAMP induces apoptotic cell death**. (A) MOPC315 cells were treated for 24 h with 200 μM 8-CPT-cAMP or 50 μM forskolin. Overall cell death, changes in mitochondrial membrane potential and DNA breaks were assessed by PI staining, JC-1 staining, and TUNEL analysis, respectively. The results are presented as mean values+/-SEM of 3 experiments. (B) MOPC315 cells (left panel) and INA-6 cells (right panel) were treated with or without 50 μM forskolin. At the indicated time points, cells were collected and subjected to western blot analysis with antibodies recognizing full length and cleaved products of caspase 9, caspase 3 and PARP. One of 3 representative experiments is shown.

To ascertain whether cAMP induces cell death by apoptosis, MOP315 cells treated with forskolin or 8CPT-cAMP were analyzed for DNA fragmentation by TdT-mediated dUTP nick end labeling (TUNEL) technique, or by analysis of changes in mitochondrial membrane potential (Δ Ψm) by staining the cells with JC-1. Forskolin and 8CPT-cAMP induced similar percentage of dead cells whether cell death was measured as percentage of cells with fragmented DNA (TUNEL assay), by changes in mitochondrial membrane potential (JC-1 staining), or by simple incorporation of PI (Figure 4A), clearly suggesting that cAMP induces apoptotic death of multiple myeloma cells.

To verify cAMP induced death of myeloma cells to be apoptoic, we investigated the downstream events following mitochondrial depolarization. Mitochondrial outer membrane permeabilization results in the release of cytochrome C from the intermembrane space into the cytosol, triggering the assembly of the caspase-activating complex that mediates autocleavage and activation of caspase 9 [32]. Once activated, caspase 9 activates downstream effector caspases such as caspase 3 provoking the cleavage of several proteins, such as PARP, which ultimately leads to cell destruction [33]. MOPC315 cells and INA-6 cells were treated with 50 μM forskolin or vehicle, and expression of cleaved caspase 3, caspase 9 and PARP were examined by western blot analysis. Forskolin induced profound cleavage of caspase 9, caspase 3 and PARP in INA-6 cells, and to a lesser extent in MOPC315 cells (Figure 4B), confirming that cAMP indeed kills human and murine multiple myeloma cells by activating the apoptotic machinery

### cAMP delays growth of multiple myeloma cells in vivo

**Figure 5:**
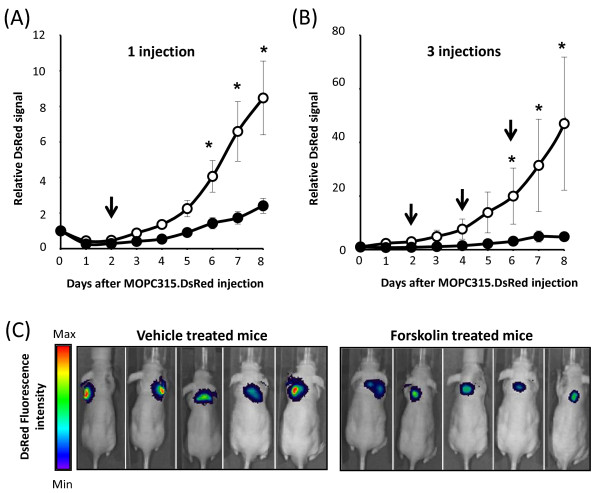
**Forskolin delays tumor growth *in vivo***. Ten BALB/c Nude mice were subcutaneously injected with 5 × 10^5 ^MOP315.DsRed cells on day 0. On day 2 (panels A), groups of 5 mice received an intraperitoneal injection of forskolin (4-5 mg/kg) or vehicle. In a separate experiment (panel B), 5 mice received 3 subsequent injections with forskolin or vehicle on days 2, 4 and 6. In vivo imaging of DsRed fluorescence from the mice was measured daily with an IVIS Spectrum detection system. (A and B) Average total DsRed fluorescence from mice (n = 5) at the indicated time points relative to day 0. Arrows indicate the time of vehicle (open circles) or forskolin (closed circles) injections. Error bars represent SEM. *P < 0.05 relative to vehicle treated mice. (C) In vivo images of individual mice taken at day 7 from the one injection experiment (panel A).

Having shown that elevation of intracellular cAMP kills multiple myeloma cells in vitro, we explored the therapeutic potential of cAMP-elevating compounds on tumor growth in vivo, taking advantage of a previously established mouse model for multiple myeloma based on subcutaneous injection of MOPC315 cells [24] pre-labeled with the fluorescent protein DsRed [34]. BALB/c nude mice were subcutaneously injected between the shoulders with 5 × 10^5 ^MOPC315 cells stably transfected with the gene encoding the fluorescent protein DsRed (MOPC315.DsRed cells). Two days after inoculation of the cells, 5 mice were intraperitoneally injected with a single dose of forskolin (4-5 mg/kg), whereas 5 mice were injected with the same volume of vehicle. Tumor size was followed daily by in vivo imaging of DsRed fluorescence using an IVIS Spectrum Imaging System from Caliper Life Sciences. All 10 mice eventually developed tumors, but a single dose of forskolin substantially delayed the tumor growth in vivo (Figure 5A). Similar results were obtained in a separate experiment where mice were injected 3 times with forskolin or vehicle on days 2, 4, and 6 after tumor cells injection (Figure 5B). Statistical differences between vehicle treated and forskolin treated mice is achieved (p < 0.05) from day 6 after tumor cell injection. Figure 5C shows in vivo images of mice taken at day 7. Together, these results suggest that cAMP-elevating compounds may indeed have a therapeutic potential in treatment of multiple myeloma.

## Discussion

We have demonstrated that intracellular elevation of cAMP levels efficiently kills both murine and human multiple myeloma cells *in vitro*, and that the cAMP-elevating compound forskolin markedly delays the *in vivo *growth of multiple myeloma cells in a mouse model.

Modulation of intracellular cAMP by directly increasing the level of cAMP in the cell or by inhibiting PDE has become an interesting approach to cancer therapy [16,35,36 for reviews]. In a phase-II study, theophylline, a methylxanthine that inhibits PDEs, proved to be effective in patients with chronic lymphocytic leukemia [37]. Activation of the cAMP pathways may either induce or inhibit cell proliferation or cell death depending on the cell type, and from our own research it is clear that the effect of cAMP also varies between different types of lymphoid cells. Thus, whereas elevation of intracellular cAMP inhibits DNA-damage induced apoptosis and p53 stabilization in BCP-ALL cells and normal B- and T cells [20], no such effects were seen in myeloma cells. It is possible that the inability of cAMP to prevent the IR-induced stabilization of p53 in myeloma cells could explain why cAMP is unable to counteract IR-mediated apoptosis in these cells.

Why myeloma cells and not BCP-ALL cells are so efficiently killed by solely elevating the level of cAMP is, however, unclear. The different players in the cAMP signaling pathway are highly compartmentalized in the cells, with G-coupled receptors, adenylyl cyclases, PKAs, Epacs, and phosphodiesterases all being brought in close proximity in distinct signalosomes within the cells [38]. It is possible that the activity of distinct signalosomes might contribute to localized, yet physiological significant differences in response to activating the cAMP signal in different lymphoid subpopulations. We also observed variations in the sensitivity to forskolin between the different myeloma cell lines used. This could presumably be due to variations in level and/or activity of the various components of the cAMP/PKA pathways in the different cell lines.

In an early paper [39], it was shown that cAMP analogs including 8-chloro-cAMP, dibutyryl-cAMP and 8-bromo-cAMP inhibited cell growth and induced cell death in glucocorticoid sensitive and resistant multiple myeloma cell lines. However, it was subsequently concluded that 8-chloro-cAMP mediated the cytotoxicity via its metabolite 8-chloro-adenosine (8Cl-AD) and not via the cAMP pathway [40,41]. Therefore, the potential for cAMP-elevating compounds in therapy of multiple myeloma was not further pursued. Recently, however, in an interesting study by Rickles and coworkers using a high throughput screening (cHTS) platform to identify new drugs to combine with existing therapeutic strategies for multiple myeloma [42], it was discovered that the agonist of the adenosine A2A receptor as well as phosphodiesterase (PDE) inhibitors synergized with glucocorticoids to inhibit cell proliferation and induce death of multiple myeloma cells [42], thereby supporting our present results.

A key finding in the present study was the novel demonstration of the ability of the cAMP elevating agent forskolin to inhibit the *in vivo *growth of multiple myeloma cells in a mouse model. It is not yet clear whether this reduced tumor growth is due to induced tumor cell death. Tumors eventually also developed in forskolin-treated mice, which could be due to the outgrowth of a small portion of forskolin-resistant cells. Attempts to give 3 doses of forskolin spaced 2 days apart did not markedly improve the effect on tumor growth compared to a single dose. A combination of cAMP-elevating compounds and conventional therapeutic agents could probably improve the outcome. The enhanced killing of myeloma cells we observed *in vitro *by combining forskolin and γ-irradiation supports this strategy. Based on the findings by Rickles et al [42], it will also be interesting to test the combination of cAMP elevating agents, phosphodiesterase inhibitors and glucocorticoids on the in vivo growth of multiple myeloma cells. It is clear that the potential for cAMP in the field of multiple myeloma is revitalized.

## Conclusion

Stimulation of the cAMP-signaling pathway not only kills human and murine multiple myeloma cells in vitro, but it also reduces in vivo growth of multiple myeloma cells in a mouse model. Elevation of cAMP kills the cells via classical apoptotic mechanisms involving mitochondrial membrane-changes and activation of caspases. These results support the potential use of cAMP elevating agents as targets against multiple myeloma.

## Competing interests

The authors declare that they have no competing interests.

## Authors' contributions

VFA designed the research, performed experiments, analyzed data and wrote the paper; POH helped designing, performing, analyzing data for in vivo research and helped in writing the paper; HH designed and analyzed in vivo imaging data and helped in writing the paper, SN helped designing the research, analyzing the data and writing the paper. AS provided material and helped in writing of the paper; RB helped designing the research and writing the paper, BB provided material, helped designing the research and writing the paper; HKB designed the research, analyzed data, and wrote the paper. All authors have read and approved the manuscript.

## Abbreviations used

BCP-ALL: B-cell precursor acute lymphoblastic leukemia; cAMP: Cyclic adenosine monophosphate; IR: Ionizing radiation; MOPC: Mineral Oil-induced Plasmacytoma; MM: Multiple myeloma; PDE: phosphodiesterases; PGE2: prostaglandin E2; PKA: Protein kinase A; PI: Propidium Iodide; TUNEL: TdT-mediated dUTP nick end labeling; 8CPT-cAMP: 8-chlorophenylthio-cAMP.

## Pre-publication history

The pre-publication history for this paper can be accessed here:

http://www.biomedcentral.com/1471-2407/11/301/prepub
